# The Power of Three: Nanomaterials for Natural Killer (NK) Cell Immunoengineering Maximize Their Potency if They Exploit Multireceptor Stimulation

**DOI:** 10.1002/adhm.202302297

**Published:** 2024-01-04

**Authors:** Helena Dodd, Nadia Guerra, Iain E. Dunlop

**Affiliations:** ^1^ Dept. Materials Imperial College London Exhibition Road London SW7 2AZ UK; ^2^ Dept. Life Sciences Imperial College London Exhibition Road London SW7 2AZ UK; ^3^ Dept. Chemistry Imperial College London Molecular Sciences Research Hub London W12 0BZ UK

**Keywords:** biomaterials, graphene oxide, immunoengineering, nanomedicine, natural killer cells

## Abstract

Many emerging cancer treatments are immunotherapies that modulate Natural Killer‐ (NK) or T cell activation, posing a challenge to develop immunoengineering nanomaterials that improve on the performance of molecular reagents. In physiological activation, multiple immunoreceptors signal in consort; however, current biomaterials do not replicate this. Here, NK cells are created for the first time, activating bionanomaterials that stimulate >2 immunoreceptors. Nanoclusters of monoclonal antibodies (mAb), templated by nanoscale graphene oxide sheets (NGO) (≈75 nm size), are exploited. To inform nanoreagent design, a model system of planar substrates with anchored mAb is first investigated. Combining mAb that stimulates three NK cell activating receptors (αNKP46 + αNKG2D + αDNAM‐1), activated NK cells act more potently than any single receptor or pair. Applying this insight, an NGO‐mAb nanocluster combining three distinct mAb: NGO‐mAb(αNKP46 + αNKG2D + αDNAM‐1) is created. This construct is potent and outperforms single‐receptor‐simulating nanoclusters, activating nearly twice as many NK cells as NGO‐mAb(αNKP46) at a similar mAb dose or delivering similar activation at 10× lower dosage. Further, NGO‐mAb are more potent than planar substrates for both single‐ and triple‐mAb stimulation. These results imply a new concept for immunoengineering biomaterials: both nanoclustering and multi‐receptor stimulation should be incorporated for maximum effect.

## Graphical abstract

Graphene oxide nanoflakes (NGO) are here used as scaffolds to create nanoclusters of monoclonal antibodies (mAb) that activate natural killer (NK) cells. The most potent activation is delivered by multifunctional NGO‐mAb that stimulate three activating receptors simultaneously. This shows that immunomodulatory biomaterials design should consider both nanoclustering and multivalent stimulation for maximum impact.

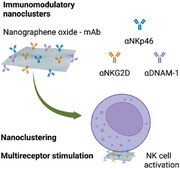

## 1 Introduction

Cancer treatments have traditionally consisted of a combination of local surgery, chemotherapy, and radiotherapy. However, as well as taxing the patient, the success of these therapies is multifactorial; hence, it can be difficult to predict. In addition, there is a need for improved methods to prevent tumor metastases and reduce the risk of cancer relapse.^[^ ^1^ ^]^ In response to these issues, new therapeutic concepts are emerging based on immunotherapies that stimulate and engineer immune cells sourced from the patient themselves or from healthy donors. Many new and proposed cancer immunotherapies directly or indirectly modulate the activation of key immune cell types, with T cells and Natural Killer (NK) cells the principal targets. For T cells, these include adoptive cell therapies based on genetically engineered activating receptors, with, for example, the first chimeric antigen receptor (CAR) T cell therapy (CAR‐T)^[^ ^2^, ^3^ ^]^ now widely approved for clinical use against hematological cancer, as well as monoclonal antibodies (mAb) such as checkpoint inhibitors that prevent cancer cells from suppressing immune responses.^[^ ^4^ ^]^ NK cells are also being widely explored as targets for immunotherapies with over 100 clinical trials of NK cell‐based cancer immunotherapy currently in development.^[^ ^5^ ^]^ These include CAR‐NK therapies,^[^ ^6^, ^7^, ^8^, ^9^ ^]^ as well as a new potential class of therapies known as multivalent engagers (MVEs) that simultaneously bind both tumor cells and immune cell activating receptors. Molecular MVEs such as: bispecific Killer cell Engager (BiKE) and Trispecific Killer cell Engager (TriKEs) constructs, consisting of single‐chain variable fragment (scFv) recombinant reagents, have been reported to enhance the specificity of NK cells toward their targets,^[^ ^10^, ^11^, ^12^, ^13^ ^]^ and a tetra‐valent approach has been reported (antibody‐based NK cell engager Therapeutic, ANKET).^[^ ^14^, ^15^ ^]^ As their names suggest, these are multi‐specific molecules, with BiKES stimulating one NK cell activating receptor in addition to binding the tumor cell, and TriKES/ANKET stimulating one or two NK cell receptors (e.g., CD16, NKp46 while simultaneously engaging cytokine receptors to promote NK cell expansion and survival^[^ ^14^, ^16^ ^]^).

Following these developments, engineering the immune response has become a key challenge in biomaterials science.^[^ ^17^, ^18^, ^19^ ^]^ This new field typically aims to address two key challenges: materials to better stimulate T cells or NK cells ex vivo for adoptive cell therapies, and the development of immunomodulatory nanomedicines such as potential nano‐MVEs^[^ ^20^ ^]^ for use in vivo. These concepts are founded on the observation that immune cell‐activation is strongly impacted by the biophysical properties of the tumor cell or activating surface, including, for example, mechanical properties,^[^ ^21^ ^]^ as well as micro‐ and nano‐spatial structure.^[^ ^22^, ^23^, ^24^ ^]^ A key observation is that most immunoreceptors and their ligands exist on cell surfaces not as isolated molecules, but rather as nanoclusters of dimensions of order 10–100 nm and containing typically a few tens of molecules.^[^ ^25^, ^26^ ^]^ In previous work, we and other labs have argued that such nanoclustering plays an important role in immune‐activation.^[^ ^22^, ^23^, ^24^, ^27^ ^]^ Further, we have exploited this insight to generate a nanomaterial‐based concept for immunoactivation, showing that mAb that activate NK cells via the CD16 receptor become more potent when they are formed into molecular nanoclusters by anchoring to a graphene oxide‐based nanomaterial.^[^ ^28^ ^]^

Here, we address receptor nanoclustering in combination with another key aspect of immune cell biophysics: the synergistic/co‐operative signaling of multiple receptors. While multi‐receptor co‐operative signaling is important in both T cells and NK cells, most T cell biomaterial studies have not explored it in detail because the combination of stimulating CD3 (part of the TCR complex) and CD28 is well‐established,^[^ ^29^ ^]^ reflecting the dominant role of the TCR complex in T cell activation. In contrast, NK cell activation quintessentially depends on integrating signals from multiple receptors.^[^ ^30^, ^31^ ^]^ This poses a challenge to the design of NK cell‐stimulating biomaterials and nanomaterials: given that a biomaterial surface can only accommodate a certain number of simulating molecules, is it best to focus on activating one or two receptors that are particularly potent or should one mimic the physiological situation by stimulating a larger number of receptors, each at a lower dose?

Here, we present a combined study of receptor‐synergy and nanoclustering in NK cell activation. Artificial molecular nanoclusters of mAb of size ≈75 nm are created by binding to a construct made from nanographene oxide (NGO), PEGylated, as we have previously shown.^[^ ^28^ ^]^ This NGO‐mAb technology can effectively provide specific stimulation to immune cells without affecting cell viability,^[^ ^28^ ^]^ and the flatness of the NGO sheets enables the sheets to reach significant size without imposing a non‐physiological curvature on the NK cell membrane. Further, NGO exhibits strength and mechanical integrity which will enable the cell apply to apply force across the nanocluster, which is important given the known role of mechanical force in NK immune cell activation.^[^ ^32^, ^33^ ^]^ However, the insights developed in this project should apply more generally to a wide range of materials used in nanomedicine, including, for example, PLGA micropatches that have been proposed as NK cell engagers.^[^ ^34^ ^]^ To create specifically NK cell‐activating molecular nanoclusters, we conjugate the NGO with mAb that bind a range of NK cell‐activating receptors: NKG2D, NKp46, DNAM‐1 and CD16. This gives the opportunity to investigate two questions in tandem: i) Is multi‐receptor stimulation more potent than single‐receptor stimulation (for the same concentration of antibodies) and ii) Is there a requirement for stimulating mAb to be nanoclustered for optimal potency? We address these questions in an in vitro model using mouse NK cells.

## 2 Results and Discussion

### 2.1 Synthesis of Mono‐ and Multivalent Nanographene Oxide‐Templated Molecular Nanoclusters

**Figure 1:**
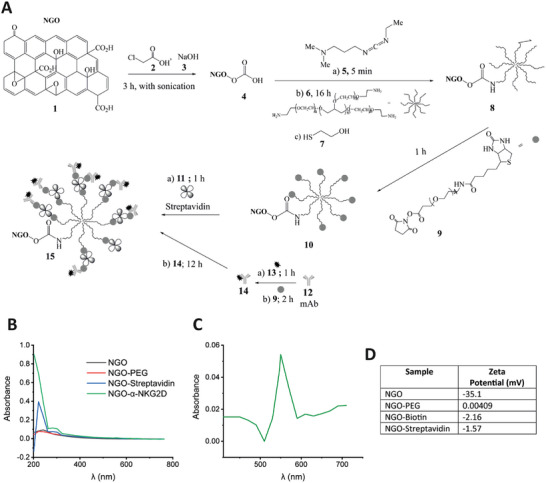
Characterisation of NGO‐based monoclonal antibody nanoclusters. The synthesis of NGO‐mAb from starting NGO was carried out stepwise, via several intermediates. A schematic of this synthesis is shown in (A). B,C) Show UV–vis characterization of some of the NGO‐based nanomaterials obtained. The appearance of protein‐characteristic absorptions including at 280 nm for NGO‐Streptavidin compared to NGO and NGO‐PEG (B). The NGO‐α‐NKG2D spectrum after subtraction of the NGO‐Streptavidin absorption spectrum (450–750 nm region only): the peak at 556 nm arises from the AF546 conjugated to α‐NKG2D (C). D) Zeta potential values in mV of NGO, NGO‐PEG, NGO‐Biotin, and NGO‐Streptavidin.

The first step was to synthesize the molecular nanoclusters consisting of nanographene oxide flakes with conjugated mAb (NGO‐mAb). This synthesis of nanographene oxide (NGO)‐templated molecular nanoclusters followed a previously‐described process.^[^ ^28^ ^]^ NGO flakes were sourced commercially, with a target size of ≈100 nm to enable the synthesis of final NGO‐mAb constructs within the broad physiological size range of immune cell receptor nanoclusters^[^ ^26^, ^35^, ^36^, ^37^ ^]^ (2D‐Tech, Manchester, UK). The functionalization of NGO to NGO‐mAb was carried out in a stepwise manner (**Figure** **1**A). First, to increase the number of available carboxylic acid groups on the sheet surface, NGO was reacted with chloroacetic acid using NaOH as an additive.^[^ ^38^ ^]^ Following this, to ensure colloidal stability of the NGO sheets in physiologically‐relevant solutions and eliminate non‐specific interactions with cells, PEGylation was carried out, using 8‐arm star‐PEG (‐NH_2_ ‐terminated arms, Creative PEGWorks) to generate a high‐density stabilizing layer.^[^ ^28^ ^]^ The reaction was enabled by activating the ─COOH groups using *N*‐(3‐Dimethylaminopropyl)‐*N*′‐ethylcarbodiimide (EDC). To enable biofunctionalization, biotin groups were added to the unreacted amine terminations on the star‐PEG‐NH_2_ using an active ester‐based linker (NHS‐PEG‐biotin). After quantification of the degree of biotinylation (HABA/avidin, see Experimental Section; Figure S1, Supporting Information), the NGO‐constructs were coated with streptavidin. Specifically, streptavidin was added in excess (10:1 molar ratio) to ensure the presence of free biotin‐binding sites and minimize cross‐linking between NGO sheets. Non‐reacted streptavidin was removed by centrifugal filtration, as was also done for unreacted reagents in the earlier phases of the synthesis. The UV–visible spectrum of the resulting construct showed protein‐characteristic peaks at 220 and 280 nm in addition to the NGO characteristic absorbance (Figure 1B).

The NK cell‐activating mAb used for this study was rat anti‐mouse NKG2D (clone MI6, eBioscience), anti‐ mouse NKp46 (clone 19A1.4), anti‐mouse DNAM‐1 (clone 10E5), anti‐mouse CD16 (clone S17014E), and IgG2aκ isotype control (clone eBM2a, eBioscience). These were fluorescently labeled and biotinylated using active ester (*N*‐hydroxysuccinimide, NHS) linkers, and reacted with the NGO‐PEG‐streptavidin constructs, with the mAb below stoichiometric ratio. As the biotin–streptavidin chemistry was common to all antibodies, the same NGO‐PEG‐streptavidin constructs could readily be linked to different individual antibody or to multiple antibodies. This approach was thus used to prepare NGO‐mAb that bind multiple receptors as well as single receptors. The binding of mAb was confirmed by UV–vis spectroscopy (NanoDrop small volume spectrometer) using the fluorescent labels, for both single‐mAb (Figure 1C) and three‐mAb (differently labeled, Figure 4A). Further qualitative confirmation of protein (mAb and streptavidin) binding to the constructs was provided by a strong absorption peak at ≈200 nm, attributable to peptide bonds.^[^ ^39^ ^]^

**Figure 2:**
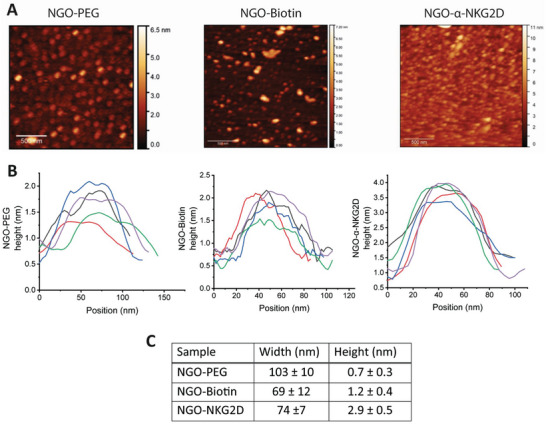
A) AFM images of NGO‐based monoclonal antibody nanoclusters. Individual AFM images obtained for NGO‐PEG, NGO‐Biotin, and NGO‐α‐NKG2D. B) Cross‐sections of typical nanosheets for NGO‐PEG, NGO‐Biotin, and NGO‐α‐NKG2D. C) Representation of a summary of the mean nanomaterial width and height values, in nm, of the three NGO constructs. Mean size values were obtained from a minimum of 40 sheets for each individual sample. See also Figure S2, Supporting Information.

**Figure 4:**
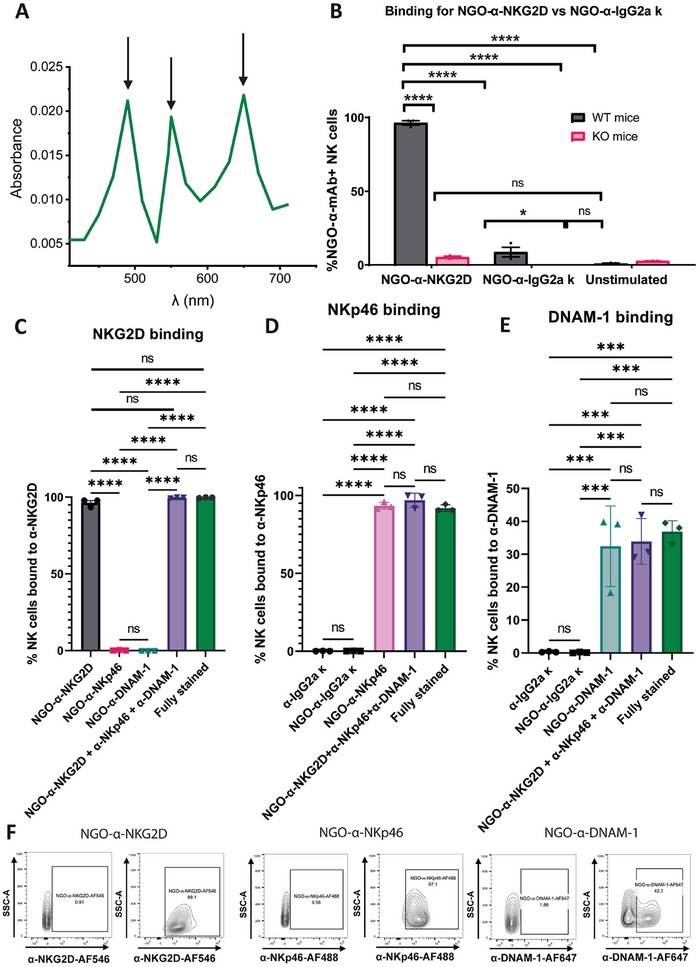
NGO‐mAb constructs bind selectively to NK cells via the targeted receptors. A) The UV–vis spectrum of NGO‐α‐NKG2D + α‐NKp46 + α‐DNAM‐1, as per Figure 1C, after subtraction of the NGO‐Streptavidin spectrum (peaks 498 nm [α‐NKp46‐AF488], 556 nm [α‐NKG2D‐AF546], and 661 nm [α‐DNAM‐1‐AF647]). B) Shows NGO‐α‐NKG2D binding for NK cells from WT versus NKG2D KO mice, measured by flow cytometry. C) NKG2D binding across other NGO‐mAb variants. D) NKp46 binding. E) DNAM‐1 binding. F) Representative flow cytometry plots from which the % binding values are extracted. One‐way ANOVA statistical analysis: ns = *P* > 0.05; * = *P* ≤ 0.05; ** = *P* ≤ 0.01; *** = *P* ≤ 0.001, and **** = *P* ≤ 0.0001

Atomic force microscopy (AFM) was used to characterize stepwise functionalization of NGO (**Figure** **2**). The sheet size for NGO‐mAb constructs was 74 ± 7 nm (mean ± standard deviation). The height of the constructs was 2.9 ± 0.5 nm: an increase with respect to the biotinylated (1.2 ± 0.5 nm) and plain PEGylated (0.7 ± 0.3 nm) precursors, indicating successful functionalization (c.f. the height of single‐sheet graphene is 0.345 nm;^[^ ^40^ ^]^ these samples are imaged dry so the height is less than if the proteins were in a hydrated state). For a detailed characterization of dispersity in a similar nanoparticle system, see our previous study.^[^ ^28^ ^]^ In parallel, the electrical charge on the constructs was evaluated by zeta‐potential measurement (Figure 1D), with NGO‐mAb exhibiting a slightly negative charge ideal for use in a biological environment. Functionalization was confirmed using UV–visible spectroscopy (Figure 1B), with the addition of both streptavidin and mAb revealed by protein‐associated adsorption peaks at 220 and 280 nm. In addition, mAb was labeled with fluorescent dyes at 488, 546, and 647 nm (Alexa Fluor dyes, conjugation using EZ‐link reagents, Fisher Scientific) before binding to the NGO constructs. The binding of mAb was confirmed by UV–vis spectroscopy (NanoDrop small volume spectrometer) using the fluorescent labels, for both single‐mAb (Figure 1C) and three‐mAb (differently labeled, Figure 4A), and calibrated absorbance curves were used to determine the concentrations of mAb and NGO. Using also the known molecular mass per unit area of NGO (1726 m^2^ g^−1^),^[^ ^41^ ^]^ the number of mAb per nanosheet was ≈122, which on the assumption that the cell will bind only one side of the sheet, gives ≈61 mAb per NGO‐templated nanocluster available for binding.

### 2.2 Optimal NK Cell Activation by Biomaterials Requires Synergetic Stimulation of >2 Receptors

To design NGO‐mAb for NK cell stimulation, we needed to determine the optimal mAb combinations that will generate potently activating nanoclusters. To achieve this, experiments were first carried out with mAb alone, without nanoclustering, attached to a planar substrate (tissue culture plastic).^[^ ^42^ ^]^ Here we addressed the question as outlined above, how best to exploit the limited number of mAb that can be immobilized on a biomaterial? As each NGO‐mAb nanocluster presents ≈61 mAb to a cell, possible strategies include using 100% identical mAb to stimulate a single potent receptor or else stimulating multiple receptors, but with lower numbers of mAb each. While NK cell receptors are known to signal co‐operatively,^[^ ^43^, ^44^, ^45^, ^46^ ^]^ the question as to whether multi‐receptor stimulation is optimal in biomaterials design has not been directly addressed to our knowledge.

We selected mAb that stimulated four NK cell‐activating receptors: NKG2D, NKp46, DNAM‐1, and CD16. These all play a role in the NK cell anti‐tumor response,^[^ ^47^, ^48^, ^49^ ^]^ and they activate NK cells via distinct signaling pathways,^[^ ^50^ ^]^ increasing the likely value of co‐operative signaling. Synergy with other receptors has been reported in biological studies for NKG2D, NKp46, and DNAM‐1.^[^ ^43^, ^44^, ^45^, ^46^ ^]^ As well as testing the activating effect of all four of these mAb individually, they were also tested in every possible pair combination, all four simultaneously, and in a triple condition using NKG2D, NKp46, and DNAM‐1. Importantly, these conditions were compared at the same overall concentration of mAb applied to the substrate; so that, any increase in activation with the multi‐receptor conditions would be attributable to co‐operativity, and not to a change in conditions.

As a cellular model for activation, we used primary mouse NK cells from wild‐type mice, with the exception of a few experiments where NKG2D‐deficient mice *(Klrk1*−*/*−) were used as negative controls.^[^ ^51^ ^]^ NK cells were purified from splenocytes by negative selection and cultured for 4 days in the presence of initial cytokine supplementation (IL‐2 (10 U mL^−1^); IL‐15 (10 ng mL^−1^).^[^ ^51^ ^]^ These cytokine levels were found sufficient to enable NK cell expansion, while also avoiding high levels of baseline activation.

To carry out activation assays, cells were plated in 96 well plates that had been pre‐treated with the relevant mAb at a total dose of 10 µg per well; so that, for example, for a pair of mAb in the well, the dose would be 5 µg of each, as outlined above; see schematic **Figure** **3**A (each well 0.32 cm^2^ surface area, 100 µL fluid volume). Cells were exposed to these stimulating substrates for 4 h, in the presence of Brefeldin A (20 µg mL^−1^) to prevent the extracellular release of IFN‐γ. The level of IFN‐γ in the cells after 4 h was used as a readout of NK cell activation and was assayed by flow cytometry after cell permeabilization. To ensure that results only reflected viable NK cells, cells were selected for analysis within the flow cytometry results by gating as (CD3−; NK1.1+) after initial size‐based gating to remove doublets and dead cells/debris (gating strategy Figure S8, Supporting Information). Activation results for different conditions are shown in Figure 3B–D with Figure 3E, 3F showing representative examples of raw flow cytometry data. Selected statistical comparisons are shown (full statistical summary: Figure S3, Supporting Information). NK1.1 stimulation is included purely as a positive activation control: it is a mouse activating receptor that is not expressed in human NK cells.

Figure 3Incubation with plate‐bound monoclonal antibodies results in NK cell activation, with a quantifiable co‐stimulatory effect detected between activating receptors. Stimulation conditions are described in the text, with A) showing a schematic illustration of the concept. Flow cytometry results show % IFN‐γ‐expressing cells as a readout of NK cell activation. Comparison of conditions is shown: B) for single receptor conditions and C) for multiple receptor conditions. D) A direct comparison of pairwise receptor conditions with individual receptor stimulation. E–H) Summary flow cytometry plots for IFN‐γ expression for all conditions in one representative repeat. All single receptor conditions (E), All receptor pair conditions (F), all triple and quadruple receptor conditions (G), and the unstimulated and α‐NK1.1 controls (H). One‐way ANOVA statistical analysis: ns = *P* > 0.05; * = *P* ≤ 0.05; ** = *P* ≤ 0.01; *** = *P* ≤ 0.001; and **** = *P* ≤ 0.0001. For full statistical comparisons between all samples, see Figure S3, Supporting Information.

**Figure:**
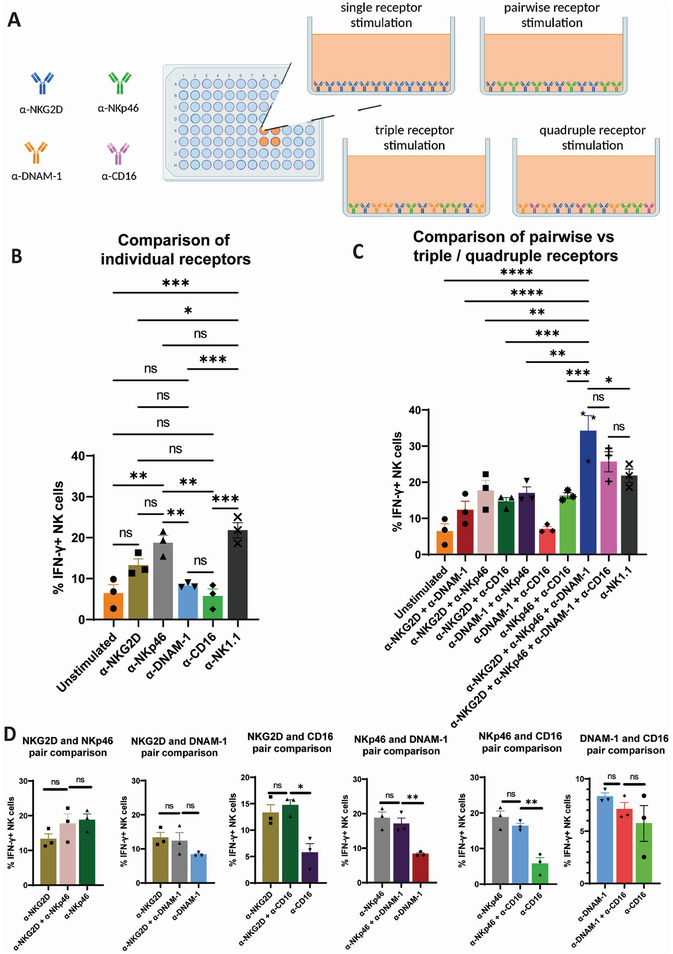

**Figure:**
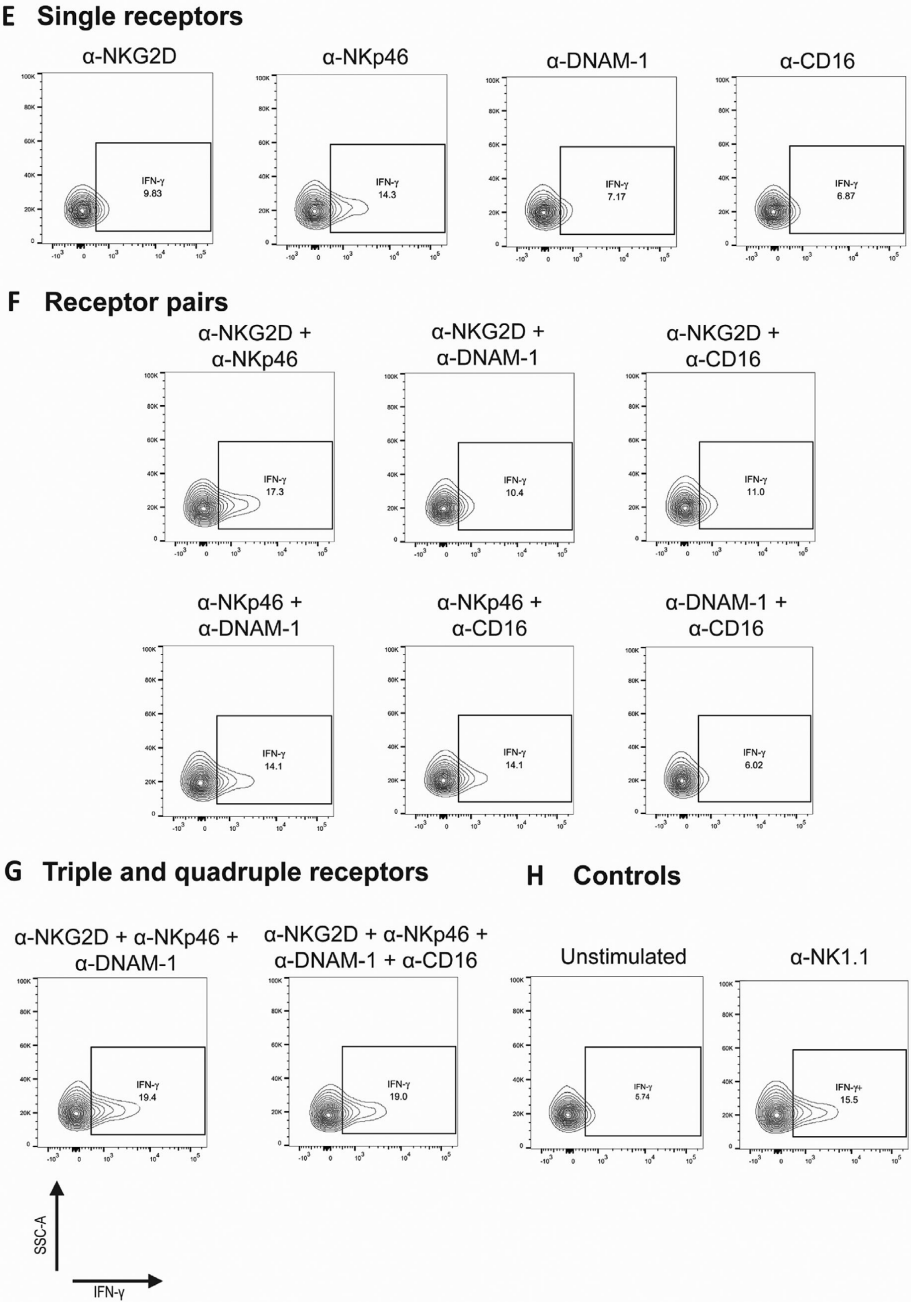

Comparing conditions that stimulate only one receptor (Figure 3B), the strongest activation comes from stimulating NKp46 or NKG2D. Hence, these would be the best receptors to select if only one receptor were to be stimulated with the biofunctionalized NGO‐mAb nanomaterials. We then consider whether better activation could be obtained by instead stimulating receptor pairs (Figure 3D). It can be seen that this is not the case. Combination with a potent activator can indeed rescue the effect of a weak activator; for example, NKp46 (5 µg) + DNAM‐1 (5 µg) is equally as potent as NKp46 (10 µg), providing evidence of synergetic signaling. However, there is no pair‐combination of mAb that stimulates more effectively than the best of the two mAb considered used separately. This suggests that stimulating two receptors will not be sufficient to offer an improved biomaterials design versus stimulating single receptors.

The picture changes when considering > 2 receptors. Specifically, the triple condition of stimulating NKG2D, NKp46, and DNAM‐1 (3.3 µg + 3.3 µg + 3.3 µg per well), and the quadruple stimulation of all four receptors (2.5 µg + 2.5 µg + 2.5 µg + 2.5 µg), both significantly outperformed all individual stimulations and all pairwise stimulations (Figure 3C). Triple and quadruple stimulation did not show significant differences from one another. We note that the activation levels under these conditions were very high, with, for example, 34% ± 6% IFN‐γ+ cells under the triple stimulation condition, versus only 23% ± 2% IFN‐γ+ for the NK1.1 condition that is widely used as a positive control and versus < 20% for any of the individual or pairwise stimulation conditions.

### 2.3 NGO‐mAb Nanomaterials Show NK Cell Receptor‐Specific Binding With High Selectivity and Do Not Impact NK Cell Viability

The results of the previous section imply that there are substantial benefits to be gained by stimulating >2 receptors when designing NK cell stimulating biomaterials. We next applied these insights to the generation of NGO‐mAb molecular nanoclusters, aiming to determine whether the benefits of multi‐receptor stimulation, as demonstrated here, and nanoclustering^[^ ^28^ ^]^ can be combined. Hence a nanotemplated version of the triple mAb stimulation condition was synthesized, consisting of NGO‐α‐NKG2D + α‐NKp46 and α‐DNAM‐1. The triple functionalization was validated using UV–visible spectroscopy with distinct dye‐labeling for each of the mAb (α‐NKG2D labelled with AlexaFluor(AF)−546 NHS ester, α‐NKp46 AF‐488; α‐DNAM‐1 AF‐647 **Figure** **4**A). In addition to this, constructs of NGO bound to each receptor individually were also synthesized for use in comparison experiments, resulting in the generation of NGO‐α‐NKG2D, NGO‐α‐NKp46, and NGO‐α‐DNAM‐1.

As a first step, specific binding of NGO‐mAb constructs to NK cells was assessed. NK cells were cultured following the same methodology used for the previous substrate‐bound mAb activation assay, and then NK cells were stimulated with NGO‐mAb constructs at a mAb concentration of 10 µg mL^−1^. Binding was assessed, using NGO‐mAb constructs with fluorescently‐labeled mAb using similarly labeled soluble mAb as controls. To ensure that any binding observed was due to receptor‐specific binding as opposed to non‐specific interference of the fluorophore or the baseline NGO sheet structure, an additional construct of NGO‐IgG2aκ was generated for use as an isotype control (Figure 4B).

Binding to the NKG2D, NKp46, and DNAM‐1 receptors was assayed for relevant NGO‐mAb constructs, with additional comparisons with unstimulated NK cells (resuspended in PBS) that were fully stained for the same receptors, using commercially available non‐templated fluorescently‐labelled mAb.

Results from NKG2D, NKp46, and DNAM‐1 receptor binding experiments are shown in Figure 4C–E and representative flow cytometry plots of this binding, compared to unstained NK cells, are shown for each receptor in Figure 4F. Results show that NK cells bind to NGO‐mAb for all four NGO‐mAb activating constructs in the same way as they bind to non‐templated mAb, with no significant differences found. In addition, binding is consistent between NGO‐mAb functionalized with one antibody compared to NGO‐mAb functionalized with three antibodies simultaneously. Experiments with NK cells isolated from *KlrK1^−/−^* mice (Figure 4B) further confirm the specificity of the NGO‐α‐NKG2D binding, with no binding observed between the NGO‐α‐NKG2D and NK cells when the NKG2D receptor is knocked out.

To assay the effect of dose and time on binding, a competing binding assay was conducted with NGO‐α‐NKG2D (Figure S4, Supporting Information). Here, cells pre‐bound to NGO‐α‐NKG2D were then counter‐stained with a different α‐NKG2D mAb of the same clone (MI‐6) but bound to a different fluorophore (PE‐Cy7). This was done to assess if, post‐NGO‐mAb binding, any receptor sites were still available to bind, or if these were all blocked. Results showed that at the lowest dose and time tested, 5 µg mL^−1^ at 4 h, NGO‐α‐NKG2D blocked ≈20% of possible additional NKG2D receptor binding, increasing to ≈45% for the highest dose and time tested, 20 µg mL^−1^ at 24 h (Figure S4B, Supporting Information).

**Figure 5:**
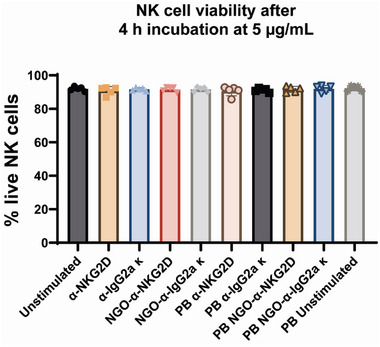
NGO‐mAb nanoclusters do not negatively impact the viability of NK cells, compared to other treatments. Figure shows %live cells after incubation with mAb and NGO‐mAb constructs, determined by staining with eFluor‐506 dye.

A viability assay was conducted to identify if the NGO‐PEG system had any negative toxicological effect on NK cell viability (**Figure** **5**). NK cells were stained with an eFluor‐506 viability dye (ThermoFisher) post‐NGO‐mAb incubation enabling a viability assessment via flow cytometry. This was conducted by first gating on NK cells, as previously described; and then, assessing the percentage of live cells that were stained with low levels of the eFluor‐506 dye, compared to dead cells stained with high levels. Results from this viability assessment show no significant difference in % live NK cells between any of the conditions tested, indicating no toxicological risk to NK cells in the context of these experiments. Further, we showed that NGO‐mAb binding did not impact the expression of the inhibitory receptor NKG2A, using again flow cytometry (Figure S5, Supporting Information).

### 2.4 Nanoclustered Templating of mAb Results in Enhanced NK Cell Activation, and This Effect is Enhanced When Combined With Receptor Co‐Operativity

We next conducted experiments to investigate whether presenting mAb to NK cells in the form of NGO‐mAb nanoclusters increased their potency, and in particular, whether this effect could be combined with enhanced stimulation due to multi‐receptor synergy. The activating effect of the nanotemplated triple‐mAb construct (α‐NKG2D + α‐DNAM‐1+ α‐NKp46) was tested, along with single‐mAb functionalized NGO‐mAb nanoclusters that bind each of these receptors. Comparison was made between NGO‐mAb and the corresponding mAb delivered in single‐molecule form.

**Figure 6:**
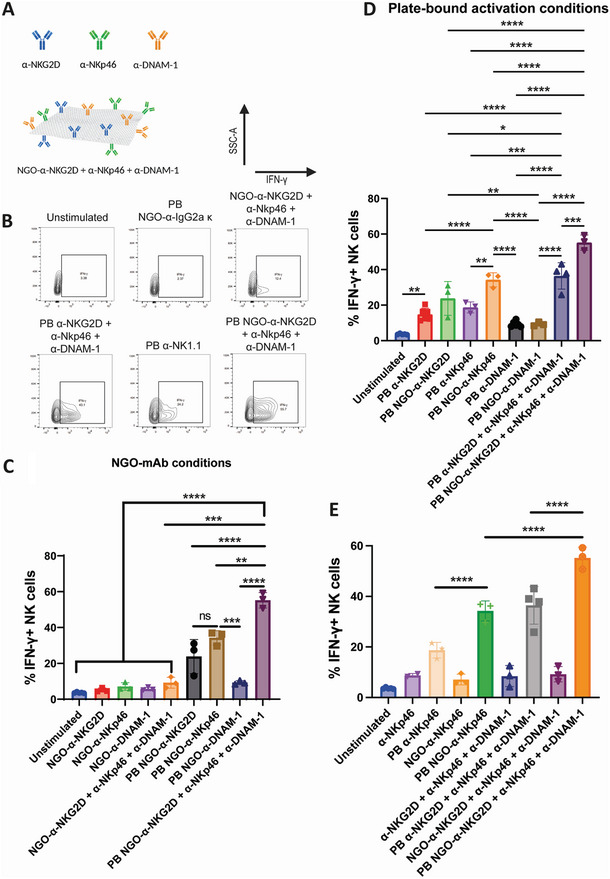
Activation of NK cells by NGO‐mAb nanoclusters and comparison of single‐mAb and multifunctional nanoclusters. A) A schematic representation of a multifunctional NGO‐mAb construct. NK cells were stimulated by mAb and NGO‐mAb constructs in plate‐bound and soluble configurations. Flow cytometry results show % IFN‐γ+ cells as a readout of NK cell activation, as follows. B) Raw data for selected conditions. NK cells were stimulated with both mAb and NGO‐mAb, with each in soluble and platebound conditions, using mAb: each of αNKG2D, αNKp46, αDNAM‐1 as well as the combination αNKG2D + αNKp46 + αDNAM‐1. C–E) Selected comparisons within these data: for a full set of statistical comparisons and full graphical representation of conditions; see Figure S6, Supporting Information. All NGO‐mAb conditions: note that all soluble conditions (in left‐hand column group) are non‐activating (with the exception of soluble NGO‐αNKG2D + αNKp46 + αDNAM‐1 which is weakly activating; see Figure S9, Supporting Information) (C). All plate‐bound conditions (D). An alternative display comparing NKp46 as the most activating single mAb, with the αNKG2D + αNKp46 + αDNAM‐1 combination, across soluble and plate‐bound formats (E). The indicated statistical comparisons show that nanoclustered mAb outperforms molecular mAb for both NKp46 and αNKG2D + αNKp46 + αDNAM‐1. In addition, the multifunctional NGO‐αNKG2D + αNKp46 + αDNAM‐1 outperforms NGO‐NKp46 and indeed all monofunctional NGO‐mAb constructs. One‐way ANOVA statistical analysis: ns = *P* > 0.05; * = *P* ≤ 0.05; ** = *P* ≤ 0.01; *** = *P* ≤ 0.001; and **** = *P* ≤ 0.0001.

Experiments were conducted using the same methodology established for the initial substrate‐anchored (plate‐bound, PB) non‐templated mAb assay to determine synergistic cooperativity effects between activating receptors. In addition to this, all conditions were tested in soluble format as well as plate‐bound, to offer a greater range of conditions for assaying the effect of mAb‐nanotemplating on NK cell activation. Isotype controls (α‐IgG2a κ and NGO‐α‐IgG2aκ) as well as unstimulated cells (suspended in PBS) were used as negative controls and plate‐bound α‐NK1.1 conditions as a positive control. NGO‐mAb and mAb‐only conditions were delivered at identical concentrations (assayed using fluorescent labeling), to allow for accurate comparison. The results of these experiments are summarized in **Figure** **6**C,D, with Figure 6A giving a schematic concept of the different conditions tested, and Figure 6B representative examples of the underpinning flow cytometry data. Selected statistical comparisons are shown here, with a full summary in Figure S6, Supporting Information.

Examining first the effect of soluble versus plate‐bound stimulation, it can be seen that stimulation with soluble NGO‐mAb is not effective in this system (Figure 6C; also Figure S9, Supporting Information). In contrast, NK cells are significantly activated by single‐mAb NGO‐mAb nanoclusters of α‐NKG2D and α‐NKp46 (but not α‐DNAM‐1), as well as the triple‐mAb construct, when these are used in plate‐bound form. This difference could be attributed to the mechanical co‐signal that is likely provided by anchoring via the tissue culture plastic (TCP) support (such mechano‐transduction has been directly observed; for example, the T cell receptor^[^ ^52^ ^]^). Alternatively, they could be due to the NK cells undergoing a basal‐apical polarization on adhesion to the TCP, such that soluble stimulation is coming via the apical direction, whereas plate‐bound stimulation addresses the cell from the basal side that more closely resembles an immunological synapse. In addition, soluble ligands will engage a monomeric receptor which is not optimum for transducing cell signaling.

We next evaluated the impact of nanoclustering by comparing the effect of molecular mAb versus NGO‐mAb, in the plate‐bound condition (Figure 6D; see also Figure 6E for an alternative visualization of selected data enabling comparison with soluble conditions). Considering the effect of stimulating NKp46, identified as the most potent stimulation above, the molecular nanoclustered NGO‐α‐NKp46 resulted in more NK cell activation compared with α‐NKp46 mAb in molecular form. This difference was substantial, with 34.3% ± 3.2% of IFN‐γ+ cells after the NGO‐mAb stimulation, compared to only 18.7% ± 2.5% for stimulation with mAb alone (*p* < 0.001). On the other hand, no statistically significant difference was observed between molecular and nanoclustered forms for DNAM‐1 or for NKG2D.

Considering the triple‐mAb nanocluster construct NGO‐ α‐NKG2D + α‐NKp46 + α‐DNAM‐1, this was found to be an extremely potent activator, with 55.2% ± 3.5% IFN‐γ+ cells. This was substantially greater than the same mAb delivered in molecular form (36.5% ± 6.4%) and also greater than the activation generated by the most potent single‐mAb construct, α‐NKp46 (34.3% ± 3.2% as previously mentioned). While experimental conditions clearly vary, we note that this is the highest levels of NK cell activation in vitro by this measure that we have been able to find in the literature, for example, refs. [46, 53, 54, 55].

**Figure 7:**
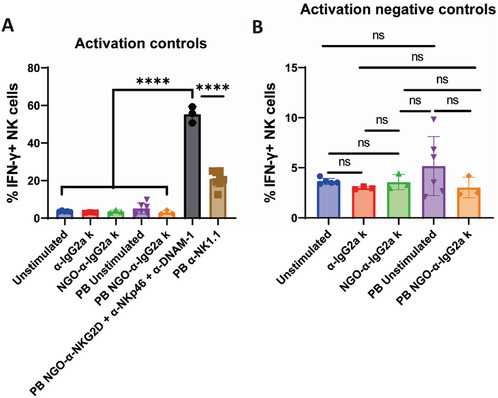
NGO‐mAb control experiments confirm no non‐specific activation by NGO‐mAb constructs with non‐binding mAb IgG2aκ. Summary of control conditions and their statistical relationships (same underpinning data as Figure 6). A) Shows all controls used and (B) only the negative controls. One‐way ANOVA statistical analysis: ns = *P* > 0.05; * = *P* ≤ 0.05; ** = *P* ≤ 0.01; *** = *P* ≤ 0.001; and **** = *P* ≤ 0.0001.

To confirm that the observed NK cell activation is due to the activating mAb themselves, and not to non‐specific effects of the NGO‐PEG construct, the results of isotype (non‐binding antibody, IgG2aκ) controls are summarized in **Figure** **7**. Stimulation with both molecular NGO‐IgG2aκ, and importantly, NGO‐IgG2aκ show no significant difference from unstimulated cells in either solubility of plate‐bound application (Figure 7B) (positive control (α‐NK1.1 stimulation) and the most potent NGO‐mAb stimulation is also included for reference (Figure 7A)). Hence, non‐specific effects are not relevant in this system.

### 2.5 The Impact of Nanoclustering on mAb Potency is Enhanced at Lower (but Viable) Doses

**Figure 8:**
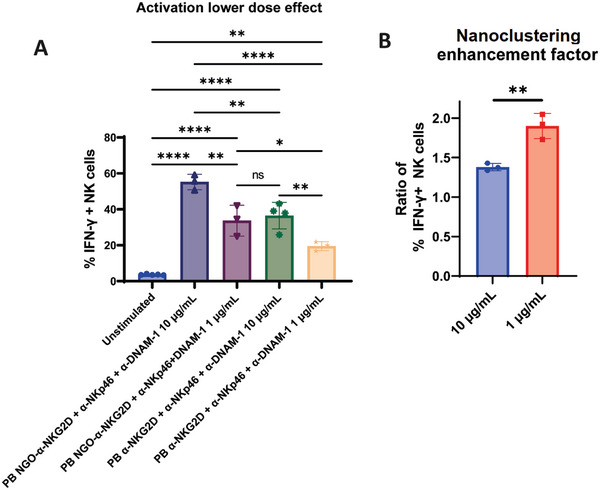
Dose effect on NK cell activating conditions. Comparison of activation experiments conducted with mAb doses of 10 and 1 µg mL^−1^. Results are summarised in (A) and the nanoclustering activation enhancement factor is shown in (B). One‐way ANOVA statistical analysis (A) and unpaired *t*‐tests (B): ns = *P* > 0.05; * = *P* ≤ 0.05; ** = *P* ≤ 0.01; *** = *P* ≤ 0.001, and **** = *P* ≤ 0.0001.

We next explored the impact on NK cell activation of lowering the mAb/nanocluster dosage, working in the /plate‐bound condition. We hypothesized that the enhancement effect due to nanoclustering should be stronger at lower dosages because the spacing between mAb on the stimulating substrate will be greater for the molecular‐mAb stimulation but unchanged for the nanoclusters. First, the mAb dose was decreased tenfold, corresponding to a dose of 1 µg mL^−1^ (**Figure** **8**A). Results from this experiment mirrored trends observed in the previous dose of 10 µg mL^−1^, with NGO‐α‐NKG2D + α‐NKp46 + α‐DNAM‐1 resulting in the greatest NK cell % IFN‐γ expression observed for any condition tested at 1 µg mL^−1^, with 33.7% ± 7.0% compared to the value of 19.5% ± 2.1% seen for the equivalent mAb only condition. Hence, the enhanced impact of nanoclustered mAb in comparison to molecular mAb is still present at this dose.

Defining a “nanoclustering‐enhancement factor” as the ratio of activation induced by NGO‐mAb compared to molecular mAb conditions, we then compared this for 10 and 1 µg mL^−1^ NGO‐α‐NKG2D + α‐NKp46 + α‐DNAM‐1 conditions. This enhancement factor, shown in Figure 8B, was found to be 1.39 ± 0.04 at 10 µg mL^−1^ and 1.90 ± 0.13 at 1 µg mL^−1^. From this, we concluded that the nanoclustering‐induced activation enhancement is indeed significantly greater at a lower dose.

In addition to the 1 µg mL^−1^ tested, a further tenfold dose reduction concentration of 0.1 µg mL^−1^ was assayed (Figure S7, Supporting Information). This was found to be too low a dose to result in any significant NK cell activation above unstimulated controls. Hence, while nanoclustering mAb can be more impactful at lower dosages, a minimum dose level is still needed for activation.

Finally, to show that the NK cells activated via the NGO constructs are able to exhibit enhanced functionality beyond cytokine secretion, we assayed the viability of tumor cells after co‐culture with NK cells that had been priorly activated using the NGO‐mAb constructs (RMA‐MULT1: lymphoma line that is further transduced with an NKG2D ligand (4 h pre‐activation of NK cells, followed by 2 h co‐culture with tumor cells) (Figure S10, Supporting Information). A reduction in tumor cell viability is indicative of NK cell cytotoxic function. Tumor cell viability was observed to be significantly reduced in the presence of NK cells that were pre‐activated using NGO‐α‐NKG2D + α‐NKp46 + α‐DNAM‐1 but not with the molecular mAb α‐NKG2D + α‐NKp46 + α‐DNAM‐1, or with isotype control NGO‐mAb. These results further confirm the potential of the multi‐functional NGO‐mAb constructs in the context of cancer applications where cytotoxic functionality is important.

### 2.6 Conclusion and Outlook

In this study, we have developed novel nanoreagents for potent NK cell activation, specifically NGO‐mAb. We have explored the impact of two key immunobiophysical phenomena: receptor nanoclustering and the synergetic stimulation of multiple receptors, using IFN‐γ expression as a measure of NK cell activation. Building on prior work, we have shown that assembling mAb into artificial molecular nanoclusters of ≈100 nm size using the NGO‐mAb technology platform renders them substantially more potent as NK cell‐stimulators, for at least some receptors (here, NKp46). Separately considering the optimal way to stimulate cells using a specified number of (substrate‐bound) mAb, we show that synergetic stimulation of multiple receptors delivers more effective stimulation than simply selecting the best single mAb. However importantly, this benefit is only seen when >2 mAb are combined.

Combining nanoclustering together with synergetic stimulation, the triple‐functionalized NGO‐mAb nanocluster NGO‐α‐NKG2D + α‐NKp46 + α‐DNAM‐1 was found to be by far the most potent stimulator of NK cells in this study. This shows that the combination of nanoclustering with synergetic stimulation of multiple receptors delivers a benefit that is not obtainable by either approach alone.

Our results have clear implications for the design of NK‐cell immunoengineering biomaterials and bionanomaterials. Biomaterials designers should in general consider stimulating a larger number of receptors simultaneously, mirroring the in vivo situation where NK cells integrate a larger number of signals. This is particularly important because it implies that NK cell activating biomaterials, whether nano‐ or larger‐scale, should not simply copy the field of T cell activating biomaterials, where a two‐receptor stimulation using α‐CD3 + α‐CD28 combination has become standard, and alternatives are little‐explored. These insights could potentially be relevant to a range of applications from ex vivo stimulation of NK cells for cell therapies to MVE development. We note the need for further development, and in particular, evaluation of the most relevant cellular outcomes, in order to assess the value of our insights in each of these specific contexts.

Considering MVEs specifically, our results show the potential value of moving beyond the BiKEs and TriKES (single‐receptor and two‐receptor stimulation) that now dominate the MVE field and toward higher valencies. They also confirm nanomaterials as a valid alternative to small biomolecule assemblies in MVE applications. Indeed, a key advantage of our (and other) nanoparticle system is that valencies can be increased without requiring new molecular technologies as is needed for BiKE/TriKE/ANKET‐style approaches. While the effectiveness of the later approaches has been extensively studied in preclinical models of cancer and shown to be safe through clinical trials, preclinical studies have shown biofunctionalized NGO‐based nanomaterials to be a promising delivery method in vivo.^[^ ^56^ ^]^

## 3 Experimental Section

### Synthesis and Characterization of NGO‐mAb

NGO was custom‐made by 2D Tech, following specifications of single‐sheet with an average and median sheet size of 100 nm. This was made from graphite following a modified Hummers’ method.^[^ ^57^ ^]^

The subsequent biofunctionalization of NGO to NGO‐mAb was carried out in a stepwise manner, as illustrated in Figure 1. Prior to the first step, carboxylation, the NGO was diluted from the as‐received 3.5 mg mL^−1^ concentration to 1 mg mL^−1^, with Mili‐Q ultrapure water. This sample was then sonicated in a water bath sonicator for 1 h in order to break up any NGO sheet aggregates. Carboxylation was then carried out following a well‐established process involving reaction of NGO with chloroacetic acid in the presence of NaOH as an additive to remove any potential oxidative residues.^[^ ^38^, ^58^, ^59^, ^60^, ^61^ ^]^ Chloroacetic acid (200 mg mL^−1^, Sigma–Aldrich) and NaOH (240 mg mL^−1^, Sigma–Aldrich) were reacted with 1 mg mL^−1^ NGO under sonication for 4 h. Excess unreacted reagents were then washed off using centrifugal filtration of the reaction mixture with centrifugal filter units (100 KDa Amicon Ultra, Merck Millipore), which involved 3× rinsing with Mili‐Q water and 5 min centrifugation at 4000 rpm. The resulting purified NGO‐COOH was then rinsed off the filter with more ultrapure water and centrifuged without a filter for 10 min at 6000 rpm for the removal of any insoluble residues. This resulted in the yielding of a stable NGO‐COOH aqueous suspension, which was brought to pH 7 by addition of a few drops of 1 mL HCl (Sigma–Aldrich).

Prior to PEGylation, the carboxyl groups now on the NGO surface were activated using *N*‐(3‐Dimethylaminopropyl)‐*N*′‐ethylcarbodiimide (EDC).^[^ ^60^, ^62^ ^]^ 8 mM EDC (Sigma–Aldrich) was added to NGO‐COOH and left to react for 5 min with stirring. Immediately following this activation, PEGylation was carried out by the addition of 40 kDa amine‐terminate 8‐arm star‐PEG (20 mg mL^−1^, Creative PEGWorks). This particular form of PEG was used because in prior work,^[^ ^28^ ^]^ the 8‐arm star showed improved colloidal stability in comparison with 4‐arm and linar PEGs. After reacting the EDC‐activated NGO‐COOH overnight with the 8‐arm star‐PEG, with stirring, the reaction was quenched with 20 mM 2‐mercaptoethanol (Sigma–Aldrich). Following the same methodology as for NGO‐COOH purification, NGO‐PEG was rinsed by centrifugal filtration, with the resulting product resuspended in PBS (Dulbecco's PBS, Sigma–Aldrich).

For the successful binding of mAb to NGO‐PEG, biotin functionality was introduced. NGO‐PEG was reacted with 0.485 mm NHS‐PEG4‐Biotin (EZ‐Link) on ice for 2 h, with shaking. The reaction mixture was then purified via centrifugal filtration, as for NGO‐PEG. Successful conjugation of the biotin onto the NGO‐PEG surface was confirmed via a HABA/Avidin assay using the Pierce Biotin Quantification kit (Thermo Fisher), following the manufacturer's instructions. UV–vis data were collected on a NanoDrop 2000 spectrometer (Thermo Fisher) to allow for very small reaction volumes. The results from this are shown in Figure S1, Supporting Information.

For streptavidin‐coating of the NGO‐Biotin, after obtaining the molar concentration of biotin in NGO‐Biotin by the HABA/Avidin assay, Streptavidin (from *Streptomyces avidinii* [Sigma–Aldrich]) was reacted with this at a 10:1 molar ratio, in excess to ensure a rapid complete coating and hence minimize cross‐linking between NGO sheets.^[^ ^63^ ^]^ The biotin‐Streptavidin conjugation reaction was left to react overnight at a temperature of 4 °C, after which unreacted Streptavidin was removed by centrifugal filtration.

Rat anti‐mouse NKG2D (clone MI6, eBioscience), anti‐NKp46 (clone 19A1.4), anti‐DNAM‐1 (clone 10E5), anti‐CD16 (clone S17014E), and mouse IgG2aκ (clone eBM2a, eBioscience) mAb were biotinylated and then fluorescently labeled before conjugation to NGO‐Streptavidin. Biotinylation was carried out as for NGO‐PEG, using 0.485 mm EZ‐Link NHS‐PEG4‐Biotin, with a HABA/Avidin assay again used to confirm conjugation. Fluorescent labeling was carried out by incubating 1 mg mL^−1^ Alexa Fluor NHS esters on ice and in the dark with the mAb separately, following the manufacturer's suggested molar ratios. Unreacted dye was removed by centrifugal filtration using 10 kDa. Successful dye conjugation was determined by UV–vis using a NanoDrop 2000 spectrometer. Alexa Fluor NHS 488, Alexa Fluor NHS 546, and Alexa Fluor NHS 647 were all used.

For the final step of the synthesis, fluorescently labeled and biotinylated mAb were added to NGO‐Streptavidin, with the concentrations calculated to ensure full binding of the mAb to the NGO sheets; so that, further purification was not needed. This also ensured that the final mAb concentration in each aliquot of NGO‐mAb was known, which allowed for NK cell mAb activation assays using the same concentration of mAb across the various conditions.

### Characterization of Functionalized NGO Nanomaterials

UV–visible spectroscopy was carried out on a VersaMax microplate reader and a NanoDrop One microvolume UV–vis spectrophotometer (Thermo Scientific). DLS and Zetasizer characterization used a Malvern Panalytical zetasizer instrument. AFM was carried out on an MFP‐3D instrument (Asylum Research), using NuNano Scout350 tips (tip radius nominal 5 nm, specified as < 10 nm). AFM samples were diluted to 1–10 µg mL^−1^ NGO; and then, drop cast onto silicon wafer cleaned with acetone, isopropyl alcohol, and deionized water washes. Tapping mode in air was used to obtain images.

### NK Cell Culturing Protocols

All animal work was carried out in designated pathogen‐free animal facilities at Imperial College London and in compliance with the British Home Office Animal Scientific Procedures Act 1986. Work was carried out under PPL licenses 70/8606 and P9718F9C8. Spleens were extracted from dissected mice and then dissociated using 100 µm MACS SmartStrainers (Miltenyi Biotec). After this, the obtained cells were suspended in ACK buffer for 1 min to lyse red blood cells, which were then removed by 5 min centrifugation at 500 g and discarding of the supernatant.

Enriched NK cell cultures were prepared by negative selection, which involved removal of other leukocytes from red blood cell lysed splenocytes using anti‐murine T cell‐specific biotinylated antibodies and streptavidin‐coated magnetic beads. 0.5% BSA in PBS was used as a staining buffer. Specifically, splenocytes were incubated for 15 min at 4 °C in an antibody cocktail containing BioLegend anti‐murine α‐CD3 clone 17A2 (5 µg mL^−1^), α‐CD4 clone GK1.5 (2.5 µg mL^−1^), α‐CD8a clone 53–6.7 (1 µg mL^−1^), α‐CD14 clone Sa14‐2 (1 µg mL^−1^), α‐CD19 clone 6D5 (2.5 µg mL^−1^), α‐Ly‐6G clone 1A8 (1 µg mL^−1^), α‐TER‐119 / Erythroid cells clone TER‐119 (1 µg mL^−1^), and α‐F4/80 clone BM8 (5 µg mL^−1^).

Centrifugal pelleting and removal of the supernatant removed excess unbound antibodies. The cell pellet was then resuspended in staining buffer and incubated for 15 min at 4 °C with MojoSort Nanobeads (BioLegend) for removal of non‐NK cell leukocytes by negative selection. Post‐incubation, the splenocytes were positioned into a 5 mL MojoSort Magnet and incubated for a further 5 min. After this, leukocytes unbound to the magnet were poured out, resulting in a now enriched NK cell population. Cells were centrifugally pelleted once more for buffer exchange and resuspension in RPMI‐1640 cell culturing medium supplemented with penicillin/streptomycin sulfate (1 µg mL^−1^), heat‐inactivated FBS (10%), L‐glutamine (2 mM), 2‐mercaptoethanol (5 µm), non‐essential amino acid solution (1 X), sodium bicarbonate (0.075%), sodium pyruvate (1 mM), and HEPES buffer (25 mM), all Sigma–Aldrich except for FBS, obtained from Gibco. Cell cultures were additionally supplemented with 10 U mL^−1^ recombinant murine IL‐2 (Peprotech) and 10 ng mL^−1^ recombinant murine IL‐15 (Peprotech) and then cultured for 4 days at 0.4 million cells per mL in a 37 °C, 5% CO_2_ incubator.

### NK Cell mAb Activation Assays

Plate‐bound mAb stimulation conditions were set up by plating mAb in a high‐adherence ELISA 96 well plate (Greiner Bio‐One). Antibodies were diluted in PBS for the desired concentration. Volumes of 100 µL per well were used for each condition. After leaving overnight to ensure adherence of the mAb to the bottom and sides of the wells, unbound mAb was rinsed off with three PBS washes.

After 4 days of culturing, NK cells were counted using a haemocytometer and Trypan Blue (Sigma–Aldrich) and resuspended at a concentration of 1 million cells per mL in RPMI cell medium, supplemented as described previously, except for the addition of cytokines. Resuspended cells were then plated into a 96 well plate at 100 µL per well. For plate‐bound stimulation conditions, NK cells were plated into previously mAb‐coated wells in an ELISA plate. For all other conditions, tissue‐culture treated 96 well plates (VWR) were used. Cells were incubated with mAb for 4 h in a 37 °C, 5% CO_2_ incubator in the presence of Brefeldin A (20 µg mL^−1^, Sigma–Aldrich), for prevention of the release of IFN‐γ.

### NGO‐mAb‐NK Cell Binding Assessment and Activation Assays

NGO‐mAbs were plated into 96 well plates at the same concentration of non‐templated mAb. NK cells were added at the same concentration of 1 million cells per mL. Cells were then incubated following the same methodology as that used for mAb conditions: for 4 h in a 37 °C, 5% CO_2_ incubator in the presence of Brefeldin A (20 µg mL^−1^, Sigma–Aldrich).

For binding experiments, NGO‐mAb was added for different time frames and at different doses. The final volume for each condition was kept consistent at 100 µL to enable accurate comparison between doses and timeframes.

### NK Cell Flow Cytometry Staining

Immediately prior to staining, cells were transferred to a 96‐well V‐bottom plate (Sigma–Aldrich) and centrifuged for 2 min at 800 × *g*. Cells were then stained for 20 min at 4 °C in the dark with 25 µL of eFluor 506 fixable viability dye (eBioscience) in PBS with 5 µg mL^−1^ F_C_ Block (clone 2.4G2, BD Biosciences), after which cells were washed with 125 µL 0.5% PBS/BSA and excess reagents were removed by centrifugal pelleting for 2 min at 800 g.

The cells were then stained for extracellular markers by resuspension in 25 µL of 3% PBS / BSA (Sigma–Aldrich) containing staining antibodies and staining at 4 °C in the dark for 30 min. In this work, the following antibodies were used: α‐NKG2D‐PE (clone CX‐5, 2 µg mL^−1^, BioLegend), α‐NKG2D‐PE‐Cy7 (clone CX‐5, 2 µg mL^−1^, BioLegend), α‐NKp46‐AF488 (clone 29A1.4, 2.5 µg mL^−1^, BioLegend), α‐DNAM‐1‐BV605 (clone TX42.1, 2 µg mL^−1^, BioLegend), α‐CD3‐AF647 (clone 17A2, 2.5 µg mL^−1^, BioLegend), α‐NK1.1‐PE‐CF594 (clone PK136, 1 µg mL^−1^, BD Biosciences), and α‐NKG2A‐PE‐Cy7 (clone 16A11. 2 µg mL^−1^. BioLegend). Washing and removal of excess reagents was carried out as before for the viability staining step.

For experiments requiring intracellular staining, cells were first fixed and permeabilized using the eBioscience Foxp3 kit. After this, cells were stained for 30 min at 4 °C in the dark with 2 µg mL^−1^ of α‐IFN‐γ‐BV421 (clone XMG1.2, 2 µg mL^−1^, BD Biosciences) in 1× permeabilization buffer (eBioscience). Finally, cells were washed once with permeabilization buffer, a second time with 0.5% PBS/BSA, resuspended in 200 µL of 0.5% PBS/BSA, and then filtered into 2 mL polystyrene tubes (Greiner) through 40 µm nylon mesh (Plastok).

### Flow Cytometry Analysis

Flow cytometry analysis of all samples was carried out using a BD LSRFortessa cell analyzer. Compensation beads were used to compensate fluorophore emission spillover. Single‐color controls were made up by staining 1 drop of negative beads and 1 drop of positive beads, (from ThermoFisher AbC Total Antibody Compensation Bead Kit), to 0.2 µg mL^−1^ mAb in 0.5% BSA in PBS. Beads were incubated for 20 min and then washed, pelleted, filtered, and resuspended in the same way as the cell samples. Compensation was carried out using BDFACSDiva software, and analysis of all samples was carried out using FlowJo version 10 software.

Statistical analysis was carried out in GraphPad Prism 9. One‐way ANOVA together with post‐hoc Tukey testing was used to compare multiple conditions.

### Additional Software

Schematic figures were produced in part using Biorender.com.

## Conflict of Interest

The authors declare no conflict of interest.

## Author Contributions

H.D. synthesized and characterized the nanomaterials and performed the NK cell experiments., I.E.D. conceived the project, I.E.D and N.G obtained the funding and supervised the research., I.E.D and N.G. designed the experiments and interpreted the data together with HD., and H.D. and, I.E.D. wrote the manuscript in collaboration with N.G.

## Supporting information

Supporting Information

## Data Availability

The data that support the findings of this study are available from the corresponding author upon reasonable request.
