# Supplementary material for: The Power of Three: Nanomaterials for Natural Killer (NK) Cell Immunoengineering Maximize Their Potency if They Exploit Multireceptor Stimulation
Source: Adv Healthc Mater. 2024 Jan 4;13(5):2302297. doi: 10.1002/adhm.202302297 (PMC11468765; doi:10.1002/adhm.202302297)
Supplement: Supplementary file 1 — Supporting Information [file ADHM-13-2302297-s001.pdf]

ADVANCED HEALTHCARE MATERIALS

Supporting Information

for *Adv. Healthcare Mater.*, DOI 10.1002/adhm.202302297

The Power of Three: Nanomaterials for Natural Killer (NK) Cell Immunoengineering Maximize Their Potency if They Exploit Multireceptor Stimulation

Helena Dodd, Nadia Guerra and Iain E. Dunlop**

The power of three: Nanomaterials for Natural Killer (NK) cell immunoengineering maximize their potency if they exploit multireceptor stimulation

Helena Dodd[1,2,3], Nadia Guerra[2#] and Iain E. Dunlop[1#]

[1]Dept. Materials, [2]Dept. Life Sciences and [3]Dept. Chemistry, Imperial College London, Exhibition Road, London SW7 2AZ, United Kingdom.

[#]Corresponding authors

SUPPORTING INFORMATION

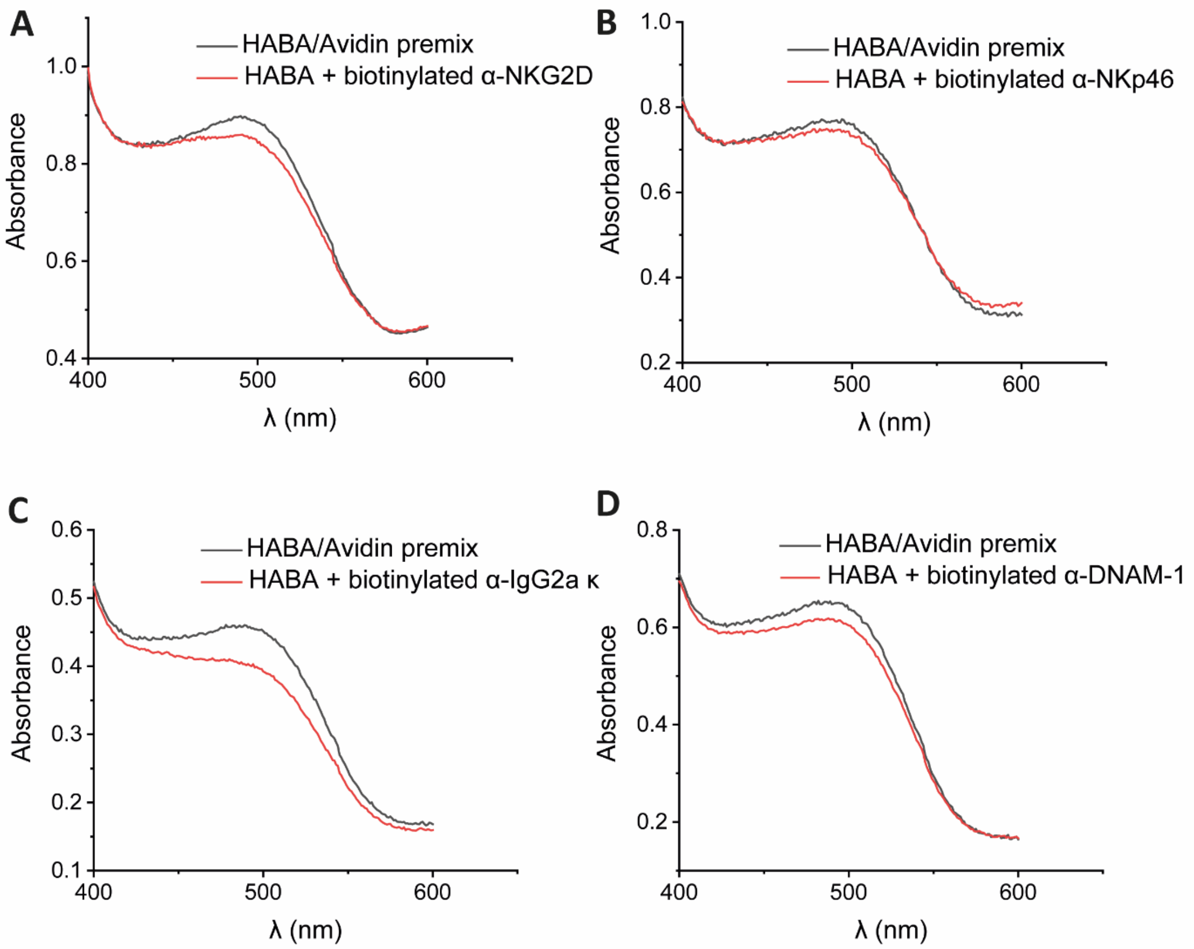

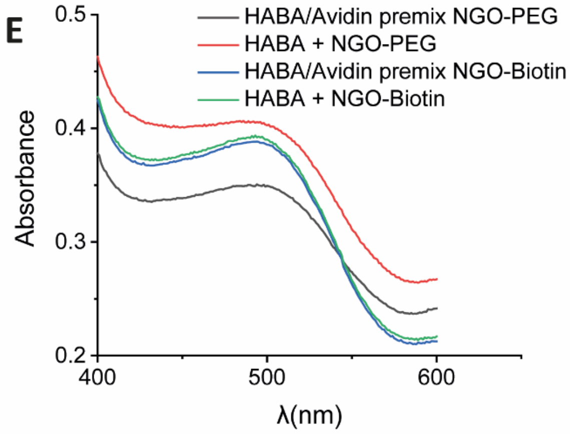

F

Sample	Biotinylation quantification
α-NKG2D	5.37 biotin molecules per mAb molecule
α-NKp46	4.70 biotin molecules per mAb molecule
α-IgG2a κ	8.37 biotin molecules per mAb molecule
α-DNAM-1	15.36 biotin molecules per mAb molecule
NGO-Biotin	1 biotin molecule every 22 nm^2

Figure S1. **Quantification of biotinylation via HABA/Avidin assay.**

Biotinylation of mAb and of NGO-Biotin was quantified via a Thermo Fisher Pierce™ Biotin Quantitation Kit, following the manufacturer's instructions. Upon successful biotinylation of a sample, the absorbance at a wavelength of 500 nm of a HABA premix decreases. This difference in absorbance can then be used to quantify the concentration of biotin present in the sample. HABA/Avidin assay results are shown for α-NKG2D (**A**), α-NKp46 (**B**), IgG2a κ (**C**) and α-DNAM-1 (**D**). Results of the NGO-Biotin biotinylation assay are shown in **E**. Quantification of biotin in this was more complex than for the mAb, due to baseline absorbance of NGO at 500 nm. To correct for this, the HABA/Avidin assay was conducted on both NGO-Biotin and NGO-PEG samples, and the difference in absorbance due to NGO-PEG was used to correct for the NGO baseline absorbance. Summary values for all biotinylation quantification are shown in **F**.

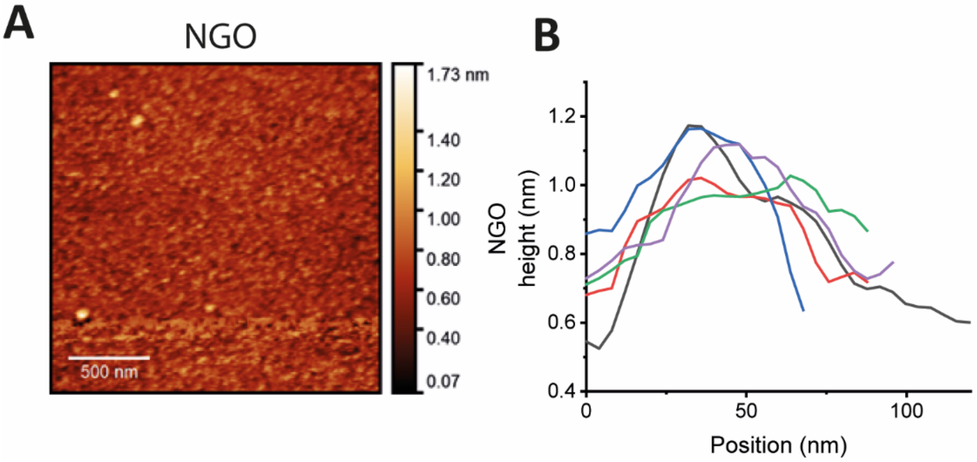

Sample	Width (nm)	Height (nm)
NGO	81.08 ± 13	0.424 ± 0.12

Figure S2. **Additional characterisation by AFM.**

AFM characterisation of NGO stock. **A** shows a topography image obtained for the NGO stock used for subsequent multi-step functionalisation to NGO-mAb. **B** shows line profiles obtained for five representative features from image **A**, with the mean NGO width and heigh values obtained in nm.

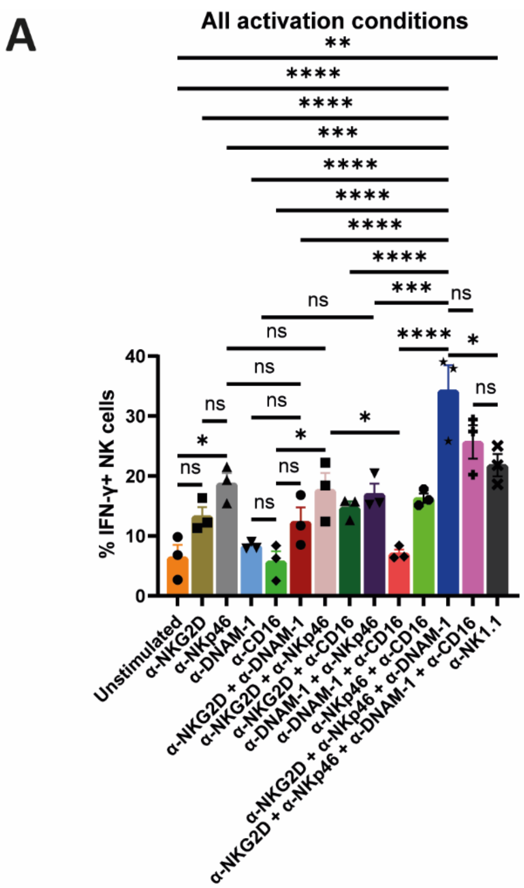

B

	Unstimulated	α-NKG2D	α-NKp46	α-DNAM-1	α-CD16	α-NKG2D + α-DNAM-1	α-NKG2D + α-NKp46	α-NKG2D + α-CD16	α-DNAM-1 + α-NKp46	α-DNAM-1 + α-CD16	α-NKp46 + α-CD16	α-NKG2D + α-NKp46 + α-DNAM-1	α-NKG2D + α-NKp46 + α-DNAM-	α-NK1.1
Unstimulated		ns	*	ns	ns	ns	*	ns	ns	ns	ns	****	****	**
α-NKG2D			ns	ns	ns	ns	ns	ns	ns	ns	ns	****	*	ns
α-NKp46				ns	**	ns	ns	ns	ns	*	ns	***	ns	ns
α-DNAM-1					ns	ns	ns	ns	**	ns	ns	****	***	**
α-CD16						ns	*	*	*	ns	**	****	****	***
α-NKG2D + α-DNAM-1							ns	ns	ns	ns	ns	****	**	ns
α-NKG2D + α-NKp46								ns	ns	*	ns	***	ns	ns
α-NKG2D + α-CD16									ns	ns	ns	****	*	ns
α-DNAM-1 + α-NKp46										ns	ns	***	ns	ns
α-DNAM-1 + α-CD16											ns	****	****	**
α-NKp46 + α-CD16												***	ns	ns
α-NKG2D + α-NKp46 + α-DNAM-1													ns	*
α-NKG2D + α-NKp46 + α-DNAM-1 + α-CD16														ns
α-NK1.1														

Figure S3. **Full set of statistical comparisons for all plate-bound mAb only co-activation conditions.**

Full set of conditions and comparisons shown, to append to **Figure 3** from the main text. One-way ANOVA statistical analysis shown, where ns = P > 0.05; * = P ≤ 0.05; ** = P ≤ 0.01; *** = P ≤ 0.001 and **** = P ≤ 0.0001

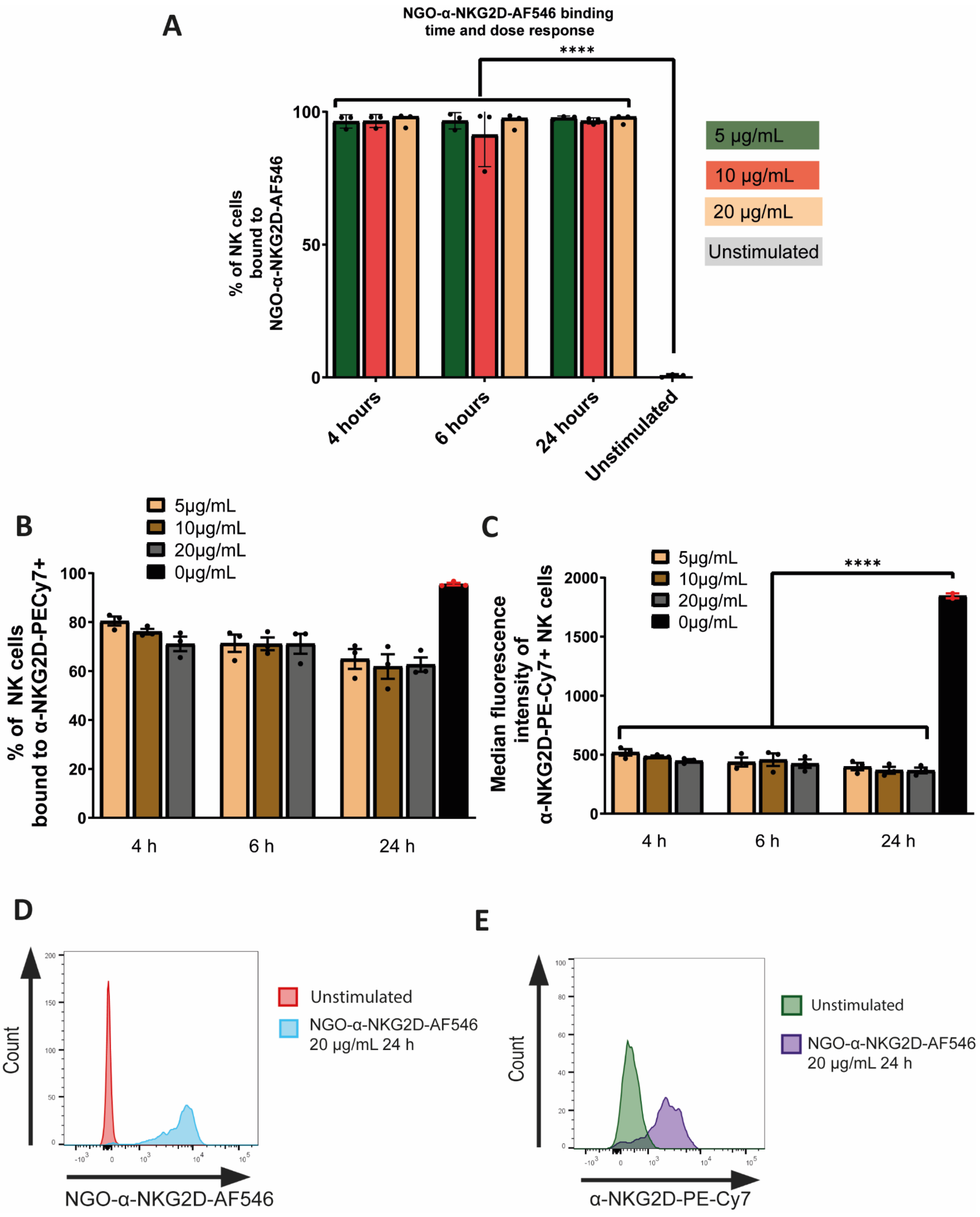
A
NGO-α-NKG2D-AF546 binding
time and dose response
****
% of NK cells
bound to
NGO-α-NKG2D-AF546
100
50
0
4 hours
6 hours
24 hours
Unstimulated
5 μg/mL
10 μg/mL
20 μg/mL
Unstimulated
B
5μg/mL
10μg/mL
20μg/mL
0μg/mL
% of NK cells
bound to α-NKG2D-PECy7+
100
80
60
40
20
0
4 h
6 h
24 h
C
Median fluorescence
intensity of
α-NKG2D-PE-Cy7+ NK cells
5μg/mL
10μg/mL
20μg/mL
0μg/mL
****
2000
1500
1000
500
0
4 h
6 h
24 h
D
Count
Unstimulated
NGO-α-NKG2D-AF546
20 μg/mL 24 h
NGO-α-NKG2D-AF546
E
Count
Unstimulated
NGO-α-NKG2D-AF546
20 μg/mL 24 h
α-NKG2D-PE-Cy7

Figure S4. Binding of NGO-NKG2D to NK cells reduces binding ability of other mAb via the NKG2D receptor, but does not fully prevent this. Day 4 NK cells were cultured in the presence of NGO-α-NKG2D-AF546 across three different doses, 5 µg/mL, 10 µg/mL and 20 µg/mL, and three different time points: 4 h, 6 h and 24 h. This was done to study the kinetic effect of NGO-mAb binding. Results from this are shown in **A**.

In addition to this, a competing binding assay was conducted, to determine the number of receptor sites still available for binding, post-NGO-mAb binding. To achieve this, cells were stained with a different α-NKG2D antibody of the same clone (MI-6) but bound to a different fluorophore, PE-Cy7. **B** shows a reduction in the % of NK cells bound to α-NKG2D-PE-Cy7 for all NGO-α-NKG2D-AF546 bound conditions, with the reduction in binding increasing from ~20% at the lowest dose and time to ~40% at the highest dose and time. **C** shows the MFI of α-NKG2D-PE-Cy7+ NK cells, where a large decrease is seen between the cells not bound to NGO-α-NKG2D-AF546 (0 µg/mL) and all cells bound to NGO-α-NKG2D-AF546. Histogram representations obtained by flow cytometry of the fluorescence of the α-NKG2D-AF546 and α-NKG2D-PE-Cy7 fluorophores are shown in **D** and **E**, as they appear in unstimulated cells (0 µg/mL NGO-α-NKG2D-AF546) compared to how they appear in cells stimulated with the highest dose and time of NGO-α-NKG2D-AF546, 24 h at 20 µg/mL.

One-way ANOVA statistical analysis shown, where ns = $P > 0.05$; * = $P \leq 0.05$; ** = $P \leq 0.01$; *** = $P \leq 0.001$ and **** = $P \leq 0.0001$

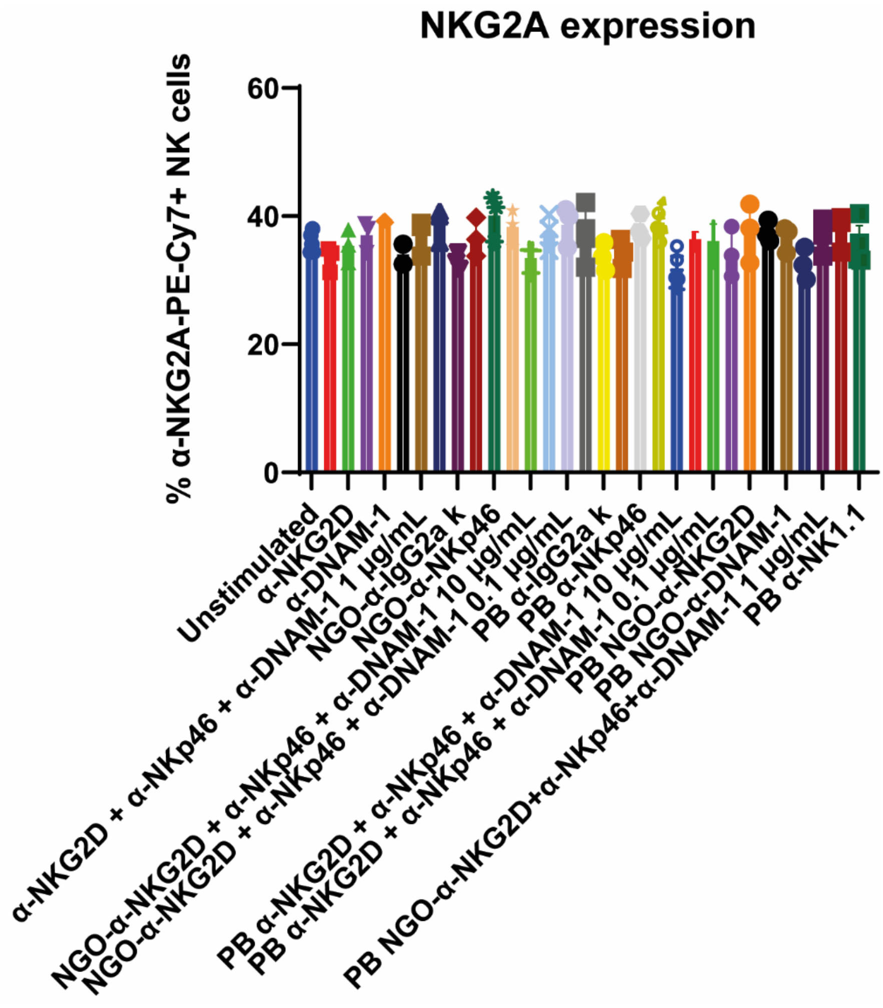

Figure S5. Effect of mAb stimulation on NKG2A expression. Post-4 h NK cell activation with 10 µg/mL PB mAb, the effect of this on NKG2A expression was studied for all conditions. A) Shows the % NK cells expressing NKG2A and B) shows the MFI of these NK cells.

A

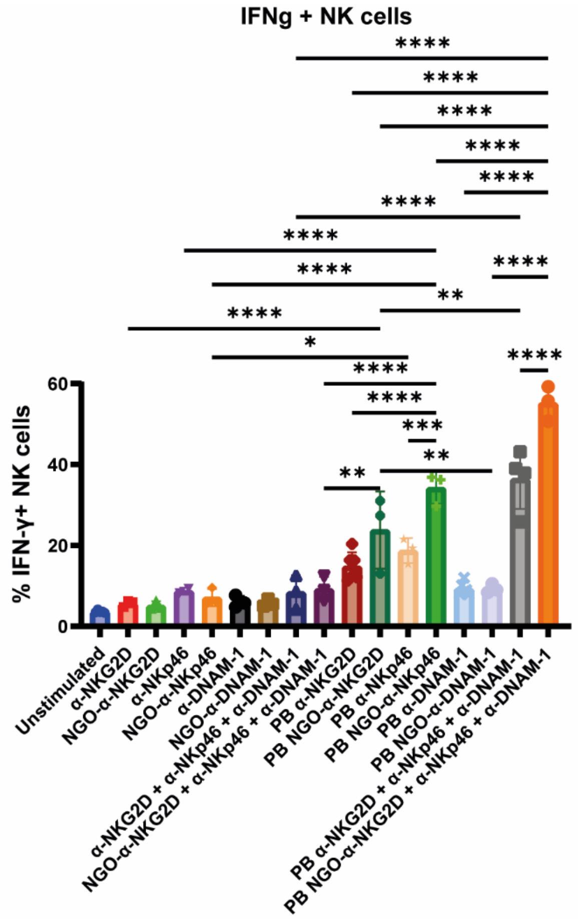

B

	Unstimulated	α-NKG2D	NGO-α-NKG2D	α-NKp46	NGO-α-NKp46	α-DNAM-1	NGO-α-DNAM-1	α-NKG2D+ α-NKp46+ α-DNAM-1	NGO-α-NKG2D+ α-NKp46+ α-DNAM-1	PB α-NKG2D	PB NGO-α-NKG2D	PB α-NKp46	PB NGO-α-NKp46	PB α-DNAM-1	PB NGO-α-DNAM-1	PB α-NKG2D+ α-NKp46+ α-DNAM-1	PB NGO-α-NKG2D+ α-NKp46+ α-DNAM-1
**Unstimulated**		ns	ns	ns	ns	ns	ns	ns	ns	**	****	***	****	ns	ns	****	****
**α-NKG2D**			ns	ns	ns	ns	ns	ns	ns	ns	****	**	****	ns	ns	****	****
**NGO-α-NKG2D**				ns	ns	ns	ns	ns	ns	*	****	**	****	ns	ns	****	****
**α-NKp46**					ns	ns	ns	ns	ns	ns	**	ns	****	ns	ns	****	****
**NGO-α-NKp46**						ns	ns	ns	ns	ns	***	*	****	ns	ns	****	****
**α-DNAM-1**							ns	ns	ns	ns	****	*	****	ns	ns	****	****
**NGO-α-DNAM-1**								ns	ns	ns	****	*	****	ns	ns	****	****
**α-NKG2D+ α-NKp46+ α-DNAM-1**									ns	ns	**	ns	****	ns	ns	****	****
**NGO-α-NKG2D+ α-NKp46+ α-DNAM-1**										ns	**	ns	****	ns	ns	****	****
**PB α-NKG2D**											ns	ns	****	ns	ns	****	****
**PB NGO-α-NKG2D**												ns	ns	***	**	**	****
**PB α-NKp46**													***	ns	ns	****	****
**PB NGO-α-NKp46**														****	****	ns	****
**PB α-DNAM-1**															****	****	****
**PB NGO-α-DNAM-1**																****	****
**PB α-NKG2D+ α-NKp46+ α-DNAM-1**																	****
**PB NGO-α-NKG2D+ α-NKp46+ α-DNAM-1**																	

Figure S6. Full set of statistical comparisons for all NGO-mAb, comparison and control conditions.

Full set of conditions and comparisons shown, to append to **Figure 6** from the main text. One-way ANOVA statistical analysis shown, where ns = P > 0.05; * = P ≤ 0.05; ** = P ≤ 0.01; *** = P ≤ 0.001 and **** = P ≤ 0.0001One-way ANOVA statistical analysis shown, where ns = P > 0.05; * = P ≤ 0.05; ** = P ≤ 0.01; *** = P ≤ 0.001 and **** = P ≤ 0.0001

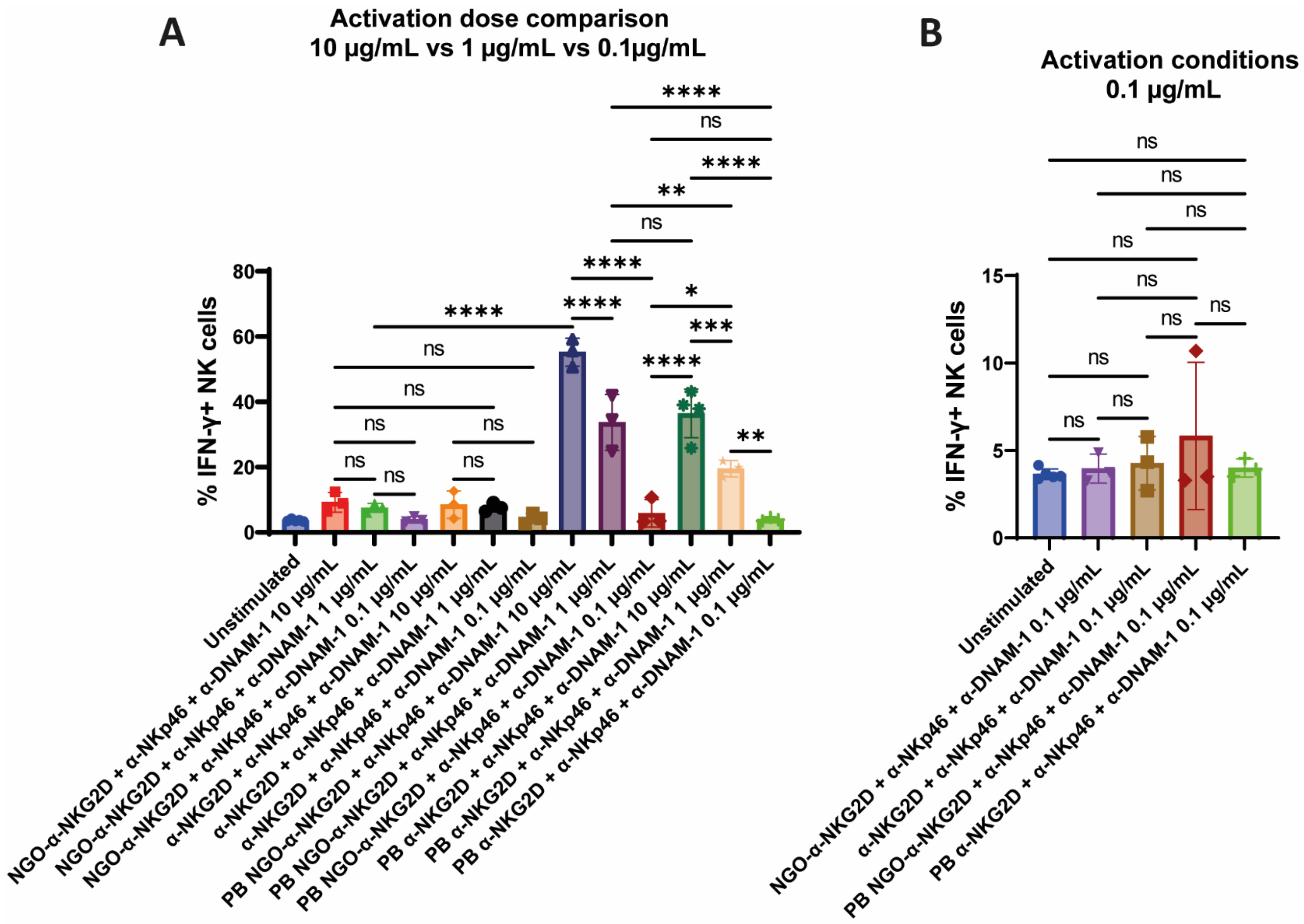

Figure S7. 0.1 μg/mL dose effect on NK cell activating conditions. Results for 0.1 μg/mL dose conditions shown, to supplement 10 μg/mL and 1 μg/mL conditions shown in **Figure 7**.

A shows a summary of all conditions, across all three doses, and **B** shows all conditions for the 0.1 μg/mL only.

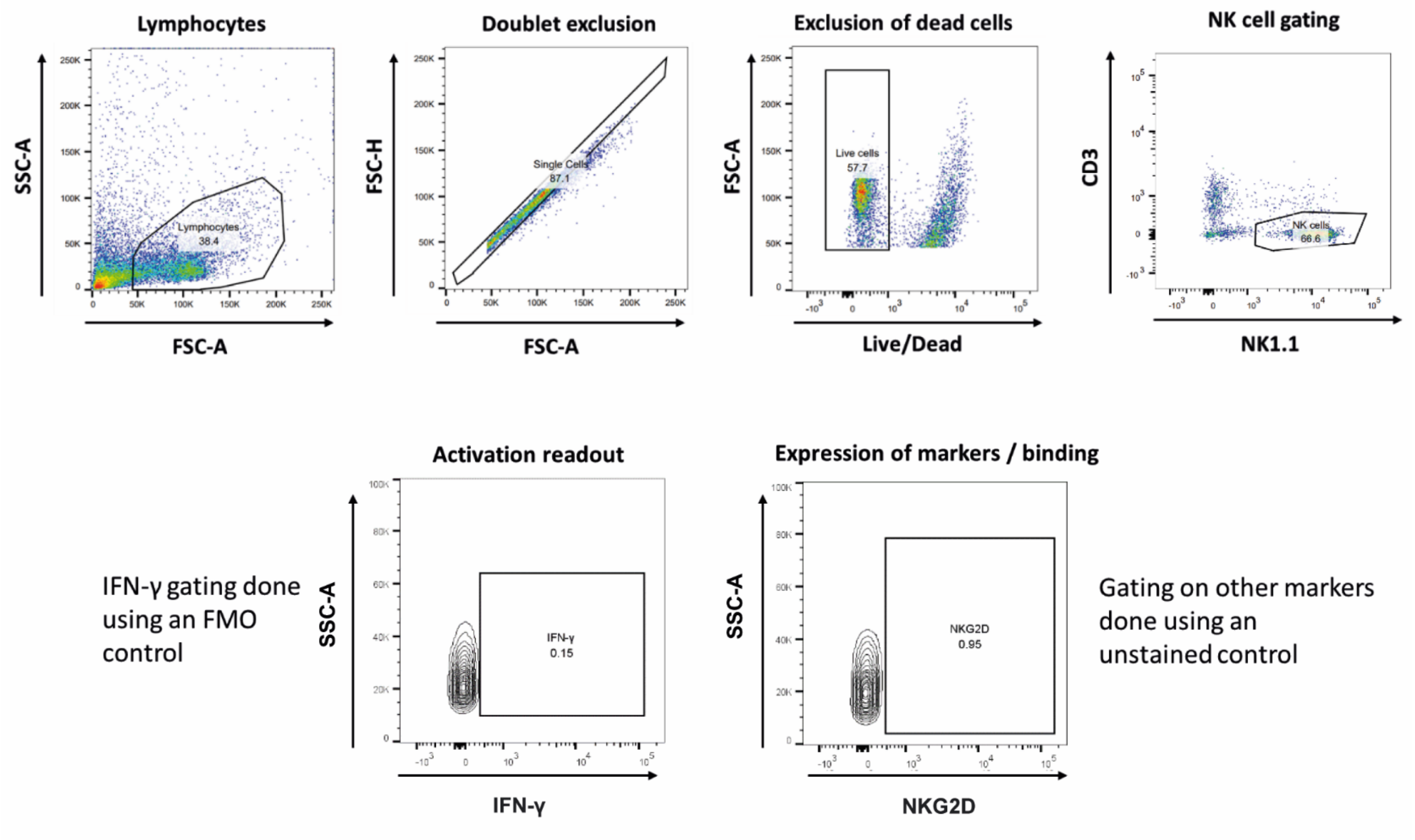

Figure S8. Standard gating strategy used for NK cell experiments. Post-culturing for 4 days, NK cells were gated as shown. First, side vs forward scatter was used to remove debris and gate for a larger lymphocyte population. Subsequently, within this gate, doublets were first excluded and then dead cells, using an eFluor-506 viability dye. After this, NK cells were selected by the NK1.1+ and CD3- population. At this point, all other markers were gated for either using FMO controls or an unstained control.

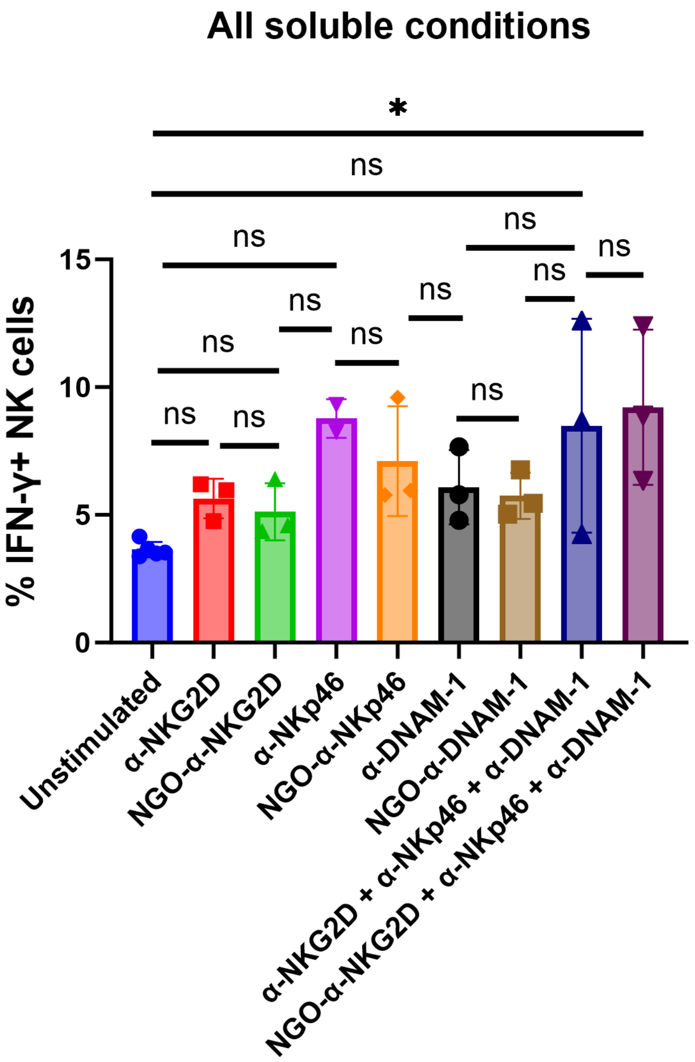

Figure S9. Soluble stimulation of NK cells by mAb and NGO-mAb. Selection of data displayed in Fig S6, illustrating soluble conditions only. All soluble conditions do not stimulate NK cells significantly in comparison with the unstimulated control, with the exception of a weak effect for the multifunctional NGO-αNKG2D + αNKp46 + αDNAM-1.

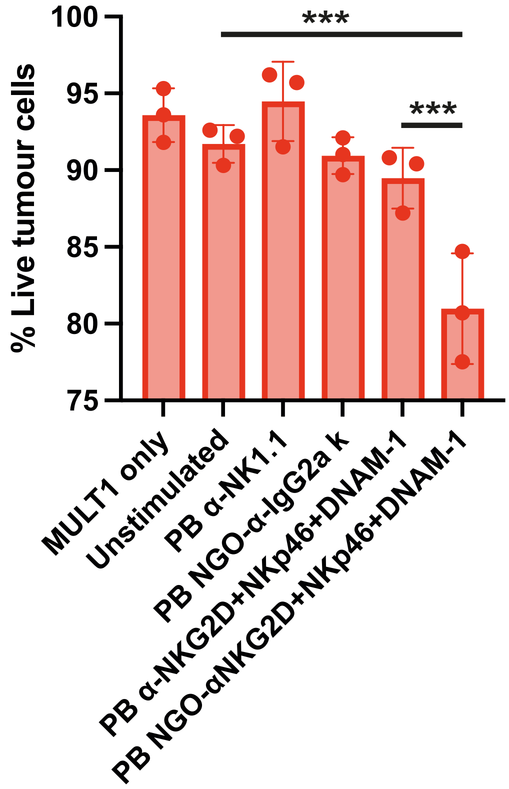

Figure S10. Tumour cell viability is reduced by exposure to NK cells that have been pre-stimulated with multifunctional NGO-mAb. Tumour cell viability is defined by %live cells. NK cells were activated with mAb and NGO-mAb for 4 hours, as before, before co-culturing with tumour cell lines for two hours, after which a viability assay was conducted. [*** $p < 0.001$, only selected comparisons shown. All stimulation conditions other than NGO-αNKG2D + αNKp46 + αDNAM-1 did not differ significantly from the unstimulated control.]

Supplementary methodology to Figure S10: The tumour cell line used was RMA-MULT1, a variant on the RMA cell line that has been transduced with an NKG2D ligand [Ref: A. Diefenbach et al. *A novel ligand for the NKG2D receptor activates NK cells and macrophages and induces tumor immunity.* Eur. J. Immunol. **33**, 381 (2003). https://doi.org/10.1002/immu.200310012]. Cells were co-cultured at a 4:1 ratio of NK cells to tumour cells Viability was determined using flow cytometry with the same staining methodology as for the viability studies described in the main manuscript. Prior to experiments, NK cells had been cultured in the same way as for other experiments described in the manuscript, including the same level of IL-2 (10 U/ml).
